# Evaluating Changes in Mental Workload in Indoor and Outdoor Ultra-Distance Cycling

**DOI:** 10.3390/sports10050067

**Published:** 2022-04-28

**Authors:** Dominic Irvine, Simon A. Jobson, John P. Wilson

**Affiliations:** 1 Epiphanies LLP, Hopyard Farm, Glanbaiden, Govilon, Abergavenny NP7 9SE, UK; 2 Faculty of Health & Wellbeing, University of Winchester, Sparkford Road, Winchester SO22 4NR, UK; simon.jobson@winchester.ac.uk; 3 Management School, The University of Sheffield, Conduit Road, Sheffield S10 1FL, UK; j.p.wilson@sheffield.ac.uk

**Keywords:** endurance, cognitive workload, EEG, theta power, NASA Task Load Index, HRV, psychomotor vigilance

## Abstract

Whilst increasing mental workload has been shown to have a detrimental effect on cycling performance and more generally to increase the risk of harm, no studies have measured how mental workload changes as a function of ultra-distance cycling, indoors or outdoors. Our objective was to measure the difference in mental workload, as indicated by changes in EEG theta power, components of HRV and psychomotor vigilance and as reported using the ‘NASA Task Load Index questionnaire’, before and after a 5 h indoor ride and outdoor ride completed at 65% of functional threshold power. Results of the NASA-TLX indicated the mental demand of outdoor cycling to be significantly less than that of indoor cycling. There were significant differences in the PVT results between the pre and the post outdoor ride average and median response times. The slowest 10% PVT responses were significantly slower pre than post the indoor ride. There were significant differences in HRV between pre and post outdoor and indoor rides, specifically, in the average RR intervals, RMSSD (ms2), LFPower (ms2), NN50. There were modest changes in indicators of mental workload during an ultra-distance cycle ride. As such, mental workload during ultra-distance cycling is unlikely to be a contributory factor to decreases in performance or to an increased likelihood of accident and injury.

## 1. Introduction

Ultra-distance cycling, where rides typically comprise many hundreds of kilometres [1], are growing in popularity, as demonstrated by a year-on-year increase in the number of scheduled events listed in the Ultracycling calendar (www.ultracycling.com, accessed on 8 January 2022) and as reported in the press [2]. The mental workload requirements of ultra-distance cycling are unclear and, whilst mental workload has been extensively explored in, for example, driving [3] and flying [4], little attention has been given to ultra-distance cycling. This matters because of the potential negative impact on performance caused by an increase in mental/cognitive workload from cycling for a sustained period of time. Increases in mental workload over time can lead to errors due to inadequate monitoring of actions [5,6], whilst also increasing the likelihood of an accident or injury [3,7].

Mental workload or cognitive workload the terms are used synonymously is “…the level of attentional resources required to meet both objective and subjective performance criteria, which may be mediated by task demands, external support, and past experience” [8]. Mental workload cannot be separated from physical effort, as one does not occur in isolation from the other [9], and mental workload is created by complex decision taking, selective attention and vigilance over time [7]. Mental workload should not be confused with mental fatigue. The focus of this study was specifically on mental workload rather than on mental fatigue. What is meant specifically by mental fatigue is problematic. Mental fatigue can occur from task underload (e.g., boredom) as well as overload ([7], and a high workload does not necessarily lead to fatigue if the rewards associated with the task are high [5,10]. Mental fatigue also depends on the perception of the task [10]. In contrast, mental workload reflects the attentional resources required to meet the demands of the task [8,11] without making any statement as to whether the participants found the experience fatiguing. Hence, the measurements used in this study are indicative of mental workload but do not infer fatigue.

Central to the concept of mental workload is the notion of limited mental resources; once the demands of a task exceed available mental resources, performance decreases [12] and task failure is more likely to occur [11]. Because the parts of the brain associated with a sense of effort are also those involved in cognitive exertion and fatigue during submaximal muscle exertion [13], the mental resources used up by mental workload reduce the capacity available for physical effort, given that attentional resources are finite [7]. For these reasons, increases in mental workload can decrease athletic performance [14,15]; hence, for the ultra-distance cyclist, increases in mental workload could lead to decreases in performance, mistakes, and increased likelihood of accident and injury.

Research investigating mental workload using cycling as the medium tends to be laboratory-based, examining performance in a secondary task following performance in a primary task [16]. For example, the distance covered in a self-paced cycling time trial was measured (primary task) before measuring performance in a Stroop Test (secondary task), where the performance in the secondary task indicated the extent to which cognitive resources were utilised by the primary task [17,18]. Typical tasks used to generate mental workload or to measure the impact of the primary task include the ‘Simon Task’ [19] and the Stroop Test [20]. These replicate neither the duration of ultra-distance cycling events, nor, prima facie, the complexity of cycling on public roads. They nonetheless provide useful insights. For example, mental workload is affected by the level of expertise in the primary task. Professional cyclists were better able to cope with mental workload demands (caused by a pre-trial Stroop Test) than were recreational cyclists in terms of the distance covered in a 20 min time trial, as reported by studies cited in [21].

In addition to experience, the duration of the primary task may also reduce the level of the mental workload experienced. For example, one of the limitations of short duration activities is that experimental sessions where the primary task lasts less than 2 h can demonstrate an adverse impact on cognitive performance, but this impact can level out and even reduce in longer duration experimental sessions [22]. If an ultra-distance cycle ride followed the same pattern, there may be no noticeable change in mental workload after an ultra-distance cycle ride, when compared to pre-ride levels.

Most research investigating mental workload in cycling is laboratory-based (e.g., [17,18]. Ultra-distance cycling events are typically outdoors on public roads or off-road on publicly accessible trails [23]. These are very different environments to the laboratory. Outdoors, riders have to cope with navigation, weather, terrain and other road users, factors which have been shown to have an effect on the mental workload experienced by drivers of motor vehicles [3,24,25]. In a laboratory setting, there are no such threats. By measuring the mental workload required for ultra-distance cycling in a laboratory setting, it may be possible to compare the results to those of mental workload involved in shorter duration cycling. By measuring the mental workload required for ultra-distance cycling in an outdoors setting, the data obtained will be more representative of ultra-distance cycling and also help determine whether the mental workload involved in ultra-distance cycling in the laboratory compares with that associated with ultra-distance cycling in a more realistic setting. Thus, the aim of this research was to both determine the changes in mental workload as a function of ultra-distance cycling in both an outdoor and an indoor (laboratory) setting. Specifically, we asked: What is the mental workload demand of ultra-distance cycling in a field setting? How does this compare with the mental workload demand of ultra-distance cycling in a laboratory setting?

## 2. Methods

We opted to use the time spent cycling at a specified level of effort as the best way of ensuring results were comparable. A riding time of 5 h was selected, as this was estimated to be the maximum duration not requiring a micturition stop if participants were encouraged. In the absence of research on urinary frequency in trained cyclists during ultra-endurance rides, the average voiding frequency rates were assumed to be between 4 and 7 times a day, i.e., approximately every 3.5 to 6 h [26]. In 5 h, participants should be expected to cover between 120 km to 145 km before needing to urinate [27].

It is not known whether ultra-distance athletes exerting different levels of effort experience different amounts of mental workload over ultra-distances. Therefore, in order to ensure a meaningful comparison both within and between participants, it is necessary to establish a common level of effort. In the absence of evidence of the typical effort required for ultra-distance cycling, the choice of effort was based on the lead author’s experience of participation in ultra-distance races, where 65% of Functional Threshold Power (FTP) [28] is used as the maximum sustainable effort over hundreds of kilometres. Bikes fitted with a power meter enable the rider to moderate their effort to sustain a consistent power output [29]. 

Thus, this study sought to (1) measure mental workload changes before and after 5 h of continuous cycling effort at 65% FTP in a field setting (i.e., on public roads) and (2) compare the response in a field setting to that observed in a laboratory setting.

### 2.1. Participants

A power calculation was performed with input variables which compared performance in a 20 km time trial following a rapid information visual processing (RVP) test with performance in a 20 km time trial without the RVP test [17]. A minimum of seven participants were required in order to achieve a power of 0.95, with an alpha error of 0.05 and a large effect size (0.65).

Eleven male cyclists (mean age 47.8, SD 6.9 years), self-declared as healthy, were recruited from the local community using snowball sampling [30,31,32]. It was anticipated that a greater number of participants than the average would drop out before completion of all the tests, given the demanding nature of the tests involved. In a meta-analysis of drop-out rates, an anticipated drop-out of 11.5% was determined [33]. All participants met the definition of a ‘trained’ or ‘well trained’ cyclist [34]. Participants were asked to refrain from exercise for 72 h prior to all tests and to avoid alcohol and caffeine for 24 hours prior to all tests [35].

### 2.2. Measures

Because of its many interrelated facets, mental workload cannot be measured directly; instead it has to be inferred from what can be measured [9]. Mental workload can be measured subjectively, for example using the NASA Task Load Index questionnaire (NASA-TLX) [36]. It can also be inferred from psychophysiological measures [7,37]. The advantage of these psychophysiological measures is that they are largely outside the control of the individual and therefore remove the subjectivity of participant perception [38].

Given the time involved, the effort expended by the participants and the context in which this study took place, four measurements were used to infer mental workload. This provided an opportunity both to triangulate the results and to mitigate the risk of one or more measurements failing. These measures were heart rate variability (HRV), theta brain wave power, psychomotor vigilance and subjective assessment, as measured by the NASA-TLX questionnaire. The choice of measures used to infer mental workload was governed primarily by the constraints of the outdoor environment in which the research took place. The following principles were applied:

1. The assessment methods cannot compromise the safety of the rider, for example obscuring their view or requiring them to forgo the use of safety equipment such as a helmet.
2. Any device used does not compromise the participant’s ability to ride their bicycle as they would normally.
3. The technology needs to have sufficient battery capacity to last the duration of each test.
4. The equipment needs to be usable in a support vehicle at the end of each of the outdoor tests. EEG, PVT and NASA-TLX cannot easily be measured whilst the participant is cycling, therefore pre and post measurement of mental workload was chosen.
5. The delay between the end of the task and data recording should be minimized to reduce the period of recovery to a minimum.

EEG, PVT and NASA-TLX cannot easily be measured whilst the participant is cycling, as trying to gather a usable EEG signal from an athlete working hard, sweating, moving along at speeds up to 50 kph on public highways whilst wearing a safety helmet was untenable. Sweat bridges across electrodes would mean the number of artifacts in the data would result in unusable data. Similar constraints apply to conducting a vigilance test on a moving participant. Had we stopped the participant mid ride, we would no longer be measuring an endurance effort but rather a series of non-ultra-endurance rides interspersed with short rest periods. Therefore pre and post measurement of mental workload was chosen. Cycling and non cycling studies (e.g., [17,21,39,40]) have demonstrated that it is possible to measure mental workload after the primary task has finished.

### 2.3. EEG

Brain wave activity, as recorded by an electroencephalogram (EEG), is the most studied indicator of mental workload [6]. There is a strong relationship between EEG frequency-domain measures of cortical activation and mental workload, specifically an increase in power in the theta band (5–7 Hz) [7,11,24,41]. Theta activity also increases with the degradation of performance as a function of boredom [7], which could be a factor in ultra-distance cycling.

EEG was recorded using a MUSE headset (InteraXon Inc., Toronto, ON, Canada), a 4-channel device: AF7, AF8, TP9 and TP10 (with reference electrode Fpz). The MUSE headset is a convenient EEG device particularly suited to ecologically valid research [42,43,44]. The muse headset has been shown to be valid and reliable [45].

### 2.4. HRV

Cardiac activity is also one of the most commonly used measures in assessing mental workload [11,46,47,48], there being much evidence to support a direct link between cardiac activity and cognitive processing [49,50]. The NN.Mean, PNN50, total spectrum power and low frequency were the key measures for detecting mental fatigue (here used as a synonym of mental workload), specifically, RMSSD was positively associated with mental fatigue, whilst PNN50 and NN.Mean were negatively associated with mental fatigue [40]. The same measures of HRV that indicate mental workload (e.g., HF, SDNN, RMSSD) also change as a function of exercise [51,52,53]. Variations in the speed of HRV recovery exist, as well-trained endurance athletes recover faster in the first few minutes than non-well-trained individuals [51]. HRV can also vary depending on the level of effort expended as a function of changes in terrain [54], hence asking well-trained participants to maintain a constant effort. It was anticipated that the impact of the exercise undertaken in the current study would induce a significant reduction in HRV. HRV recovery is delayed after prolonged exercise duration [53]. As a result, a change in HRV is not necessarily indicative of an increase in workload but, when taken in conjunction with changes in theta brain wave power, vigilance and the results of the NASA-TLX, it will demonstrate whether ultra-distance cycling induces mental workload.

Concurrent with the EEG recording, HRV data were collected following the protocol outlined by [55]. Breathing was paced using a visual cue at 0.1 Hz using the ‘Breathe+’ app Breathe+ Simple Breath Trainer (Dynamic App Design LLC, www.dynamicdesign.com, accessed on 8 January 2022), whilst seated, with data collected for 5 min. The MUSE headset was set up in approximately 2 min, keeping to a minimum pre-data collection post ride recovery. HRV was captured using a Cardiosport TP5 chest band (Cardiosport, Waterlooville, Hampshire, UK), and the data was recorded on a Garmin 1030 head unit (Version 9.50; Garmin, Schaffhausen, Switzerland).

### 2.5. Vigilance

One element of cognitive performance is being able to maintain sustained focus on a task, referred to as vigilance [56,57]. Vigilance is measured in terms of reaction times and/or the number of errors made, which are referred to as vigilance decrement [58,59]. Measures of fatigue include lapses, where the participant fails to respond within 500ms, the average reaction time, and the fastest and slowest 10% of reaction times [60]. The method used for measuring vigilance was a psychomotor vigilance test (PVT) [61]. Vigilance is affected by circadian modulation [61] and a lack of sleep [62,63,64]. Psychomotor vigilance was recorded using the ‘Vigilance Buddy’ app (Research Buddies, Apple App Store) on a 6th generation iPad (Apple Inc., Cupertino, CA, USA). A touch screen psychomotor vigilance task was assessed and validated [60,65]. Participants were instructed to use the index finger of their dominant hand, whilst holding the iPad in landscape mode to tap the screen as soon as the stimuli appeared. The stimulus was a timer that counted up in milliseconds from 0 and stopped once the screen had been touched. The time at which the screen was touched was displayed for 1s. The maximum gap between each presentation of the stimulus was 3500 ms and the minimum was 500 ms. The duration of the test was 5 min.

### 2.6. NASA-TLX

The ‘gold standard’ of subjective measures of mental workload is the NASA-TLX [24,36,66,67,68,69]. NASA-TLX correlates significantly with cardiovascular and neurophysiological indicators of mental workload. The suitability of the NASA-TLX was used to assess mental workload in sport, specifically in swimming, although the duration of the test was considerably less than that of ultra-distance cycling [68].

The participants completed a NASA-TLX questionnaire on the iPad using the NASA-Task Load Index app (Human Systems Division, NASA, United States Government). Moroney et al. (1992, cited in [70]) demonstrated that a 15-minute delay between the task and test does not significantly interfere with the ratings given by participants. For this reason, the questionnaire was completed following the collection of HRV, EEG and PVT data, completion of which took ~15 min once the participant had completed each of the cycling tests.

### 2.7. Cycling Conditions

**Figure 1:**
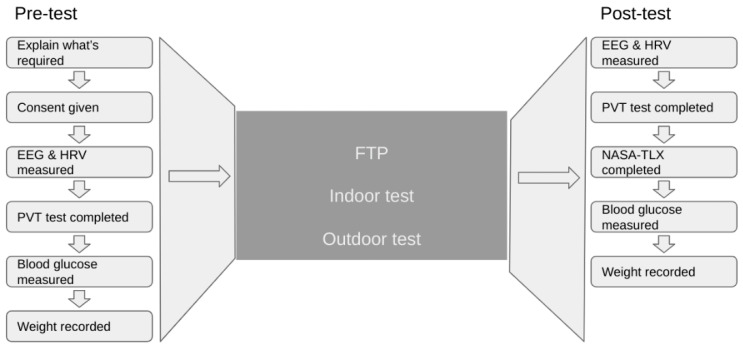
Sequence of activities before and after the test.

On their first session, on completion of EEG, HRV and PVT measurements, all participants completed an FTP test. This was followed by measurement of EEG, HRV and PVT and, in addition, the completion of a NASA-TLX questionnaire. The FTP result was used to determine the watts at which the subsequent sessions would be completed, and the other measurements were conducted to familiarize the participants with the procedure. In their second and third sessions, EEG, HRV and PVT measurements were taken, and then the participants completed either a 5 h continuous indoor ride at 65% of FTP (indoor ride) or an outdoor continuous 5 h ride at 65% of FTP (outdoor ride) followed by the measurement of EEG, HRV and PVT and the completion of the NASA-TLX questionnaire. The second and third sessions were performed in a counterbalanced order after random designation of the first participant. See Figure 1 for a summary of the process.

For the FTP test and the 5 h indoor ride, the bike was attached to a Wahoo Kickr Smart Trainer (Wahoo Fitness, Atlanta, GA, USA) set in resistance mode. Both the FTP test and the indoor ride were controlled using the app ‘TrainerRoad’ (Peaksware LLC, Boulder, CO, USA) loaded onto the iPad, with the screen, visible to the participant, displaying actual, average and target power, cadence and heart rate.

Up to three cooling fans were available for participants, if required. Reliable and valid power data over a broader and more appropriate range than the Kickr can be achieved using PowerTap P1 pedals; hence these were chosen. The PowerTap P1 pedals were validated [71,72,73] against a Scientific SRM system, and it was concluded they are a valid and reliable device to measure power output in cyclists, at power levels between 150 w and 350 w, with a cadence of 70 rev·min^−1^ [71,72]. This range of power covered the range of FTP of the participants.

Science In Sport (SIS, London, UK) energy bars, gels, energy drink and water were available ad libitum. These were placed next to the participant during the indoor ride, the gels and bars were placed in a bag on the top tube of the bike and the bottles in bottle cages for the participants during the outdoor ride. The participants were encouraged to consume between 60 and 90 g of carbohydrates per hour to ensure they were adequately fuelled throughout the ride. Blood sugar levels provide an indication of whether the participants had consumed adequate energy and were measured pre and post all tests using a Braun Omnitest 3 (Proctor and Gamble, Cincinnati, OH, USA). The participants were weighed pre and post-test, and the differences compared to provide an indication of hydration maintenance. All participants consumed the required amount of energy and fluid [73].

For the FTP test and the indoor ride, the participants followed a predefined session using Trainer Road. In this software, a workout can be programmed for a specific duration at a designated power output. TrainerRoad connects via Bluetooth to the Wahoo Kickr, PowerTap P1 pedals, Cardiosport heart rate strap and cadence sensor and displays the data received from each device.

The indoor ride included a 5 min warm up to the target power output. During the outdoor ride, the mean power output and actual power output were displayed on a Garmin 1030, alongside a map of the route. The outdoor ride route was 45 km, with 366 m of ascent. The participants cycled around the loop multiple times until 5 h of total riding were completed. At this point, a safe place was identified, and the participant stopped, dismounted from their bike and were seated in a vehicle. HRV, EEG, PVT, NASA-TLX, blood glucose and body mass were measured. Participants used their own bike for all tests.

The start point for the outdoor test was also the location for the FTP test and indoor ride. The test location was a well-lit, non-airconditioned room, maintained at a minimum of 15 °C. The walls were plain white, and the windows were translucent. A minimum rest period of 72 h was required between each test to allow time for adequate recovery [74]. The gap between the trials was on average 10 days. In addition to establishing each participant’s FTP, attendance for the FTP test was used as an opportunity to familiarize the participants with the experimental procedure.

Other than the iPad, no other electronic devices or music were available to the participants during the rides, and conversation with the participants was avoided. Because PVT is affected by circadian modulation [62], the tests started between 8 a.m. and 12 p.m. The participants reported the perceived quality of the previous night’s sleep on a scale of 1 to 5, where 1 was “slept very badly, hardly at all” and 5 was “great night’s sleep, feeling very rested.” No participant reported a score lower than 3.

### 2.8. Statistical Analysis

Descriptive statistics are reported as means and standard deviations; 95% confidence intervals of the differences between post and pre indoor ride and outdoor ride were calculated to detect significant changes.

EEG data were analysed using EEGLAB (Swartz Center for Computational Neuroscience, 9500 Gilan Dr, Dept 0523, La Jolla, CA 92083, USA). Bandpass filters <1 Hz and >30 Hz were applied, as was a notch filter at 50 Hz. Artifact rejection was set at 75 microvolts. The power for theta band was calculated using Fast Fourier Transformation (FFT). The obtained power values were averaged across the data collection period [6,41]. In the analysis of the PVT results, reaction times ≤100 ms (less than the anticipation time) were excluded from further analysis for each participant. Reaction times exceeding 500 ms were treated as lapses. EEG, HRV and PVT were analysed by applying a two-way (22) repeated-measures ANOVA to examine the differences between pre and post ride and outdoor and indoor ride. The Huynh–Feldt correction was applied. The statistical analysis was conducted using SPSS software, version 27.0. A 5% significance level was adopted in all tests.

## 3. Results

Body mass was a little lower at the end of the tests than it was at the beginning (mean change in weight [pre indoor ride − post indoor ride] = 0.4 kg, SD = 1.25; mean change in weight [pre outdoor ride post outdoor ride] = 0.7 kg, SD = 0.82). Blood glucose increased by the end of the test (mean change in blood glucose [pre indoor ride − post indoor ride] = −2.34 mmol, SD = 1.72; mean change in blood glucose [pre outdoor ride − post outdoor ride] = −1.4 mmol, SD = 2.16). The average distance covered in the outdoor test was 127.2 km. Whereas the indoor test involved no stopping, during the outdoor ride, the average time stopped (at junctions or road works) was 25 s, during an average ride duration of 04:59:34. The variation from the required effort by the participants was −0.2 W for the indoor ride and −0.7 W for the outdoor ride. This represents 0.4% variation from the average target power output. There was no significant difference (*p* = 0.05) between the average power in the indoor ride and that in the outdoor ride (mean outdoor ride power 169.1 W ± SD 40.3; mean indoor ride power 168.5 W ± SD 40.7).

There was a significant decrease in the mental demand component of the NASA-TLX in the outdoor session compared to the mental demand in the indoor session (outdoor ride mean = 164.55 a.u., SD = 114.94; indoor ride mean = 319.55, SD = 125.60, t(10) = −3.231 *p* < 0.009).

One participant stopped once during the indoor ride to micturate. Another participant stopped three times during the indoor ride to micturate. Analysis of the results revealed for the second of these participants an indoor frustration score on the NASA-TLX of 400. This score contrasts with that of all other participants, for whom the average was 5. Another participant failed to complete their first indoor ride attempt (possibly due to inadequate intake of energy), completing it later in a second attempt, after which a very low mental demand score on the indoor ride (70) was recorded, compared to that of the remaining participants, for whom the average was 344.5. This suggests that familiarisation with the task may have helped with the perceived mental demand of the task. When participants 5 and 9 were excluded from the analysis, there was a significant decrease in mental demand in the outdoor session, compared to the mental demand in the indoor session (outdoor ride mean = 195 a.u., SD = 102.9, indoor mean = 337.33, SD = 105.24, t(8) = −2.672 *p* = 0.028). This suggests that indoor cycling was perceived as more mentally demanding than outdoor cycling. There were some differences recorded, specifically for the temporal, performance, effort and frustration scores on the NASA-TLX questionnaire, which were lower for the outdoor ride than for the indoor ride. In contrast, physical demand was greater for the outdoor ride than for the indoor ride. Taking all dimensions of the NASA-TLX together, there was a significant decrease in the weighted average in the outdoor session compared to the weighted average in the indoor session (outdoor ride mean = 48.88, SD = 18.44; indoor ride mean = 64.85, SD = 11.95, t(10) = −2.378 *p* = 0.039).

There were no significant interactions between outdoor/indoor ride and pre/post ride for any of the measures. For theta power, there was a significant main effect pre and post ride: F(1,10) = 4.97, *p* = 0.05. For components of HRV, there were significant main effects pre and post ride for the following: average RR intervals F(1,8) = 165.55, *p* = 0.000. RMSSD(ms2) F(1,8) = 10.06, *p* = 0.013. NN50 F(1,8) = 26.92, *p* = 0.001. LFPower(ms2) F(1,8) = 5.57, *p* = 0.046. LF:HF Ratio F(1,8) = 21.21, *p* = 0.002. There was no significant effect for Total Power. In the PVT results, there were significant main effects pre and post ride for the following: median score F(1,10) = 11.28, *p* = 0.007, slowest score F(1,10) = 5.71, *p* = 0.038, slowest 10% F(1,10) = 9.58, *p* = 0.011.

Subsequent post hoc analysis suggested an increase in theta power post indoor session (mean = 3.8, SD = 1.05) compared to pre indoor session (mean = 3.38, SD = 1.81, but this was not significant, t(10) = 0.665, *p* = 0.052). There was an increase in theta power post outdoor session (mean = 4.43, SD = 1.73) compared to pre outdoor session (mean = 3.18, SD = 0.72). The difference, however, was not significant (t(10) = 2.219 *p* > 0.051). There were significant differences in the average slowest 10% of responses in the PVT between post indoor ride and pre indoor ride t(10) = −3.992, *p* = 0.003. There were significant differences between post outdoor ride and pre outdoor ride responses for the average PVT response, t(10) = −2.708 = 0.022, and the median PVT response, t(10) = −3.166, *p* = 0.01. There were significant differences pre versus post indoor in the slowest 10% of responses t(10) = −3.992, *p* = 0.003 t(10) = −3.992, *p* = 0.003.

For HRV, there were significant results post and pre ride for both the indoor and the outdoor rides. Post versus pre indoor ride: mean RR versus pre indoor ride RR t(7) = −27.39, *p* = 0.000; RMSSD (ms2) t(7) = −4.54, *p* = 0.003. NN50 versus t(7) = −3.80, *p* = 0.007; LF Power(ms2) t(7) = −4.07, *p* = 0.005.;Total Power (ms2) t(7) = −3.81, *p* = 0.007. Indoor versus outdoor pre-ride LF/HF ratio t(7) = 2.95, *p* = 0.022. Post versus pre outdoor ride: mean RR versus pre indoor ride RR t(7) = −22.52, *p* = 0.000; RMSSD (ms2) t(7) = −3.98, *p* = 0.005. NN50 versus t(7) = −4.04, *p* = o.005; LF Power (ms2) t(7) = −3.13, *p* = 0.017; Total Power(ms2) t(7) = −2.91, *p* = 0.023. There were no significant differences between the indoor and outdoor post-ride measures.

## 4. Discussion

Both 5 h indoor rides and outdoor rides generated significant differences in physiological indicators of mental workload. The changes in HRV would be expected as a function of exercise, as well as mental workload, and so these significant changes alone are insufficient evidence of mental workload. The decrease in the slowest 10% of responses in the post indoor and outdoor rides indicates some increase in mental workload as does the decrease in the median response in the post outdoor ride compared to the pre outdoor ride. However, when taken in conjunction with the lack of changes in theta power, the conclusion is that the increase in mental workload was slight. Given these modest changes, the implication for the ultra-distance cyclist is that mental workload is unlikely to be a contributory factor to decreases in performance or to an increased likelihood of accident and injury. The perception of higher mental workload during the indoor ride, as measured using the NASA-TLX, was not matched by physiological changes recorded through EEG, theta power or PVT, as there were no significant differences between the outdoor rides and the indoor rides in these variables. These findings do not replicate research investigating shorter duration cycling efforts, e.g., 20 km time trials [17]; however, they are more in line with the findings that cognitive performance may reach a steady state or even improve over a 2 h period [22].

The perception of greater mental workload indoors when compared to outdoors, as indicated in the NASA-TLX data, may be explained by the attention restoration effect due to the natural environment in which the outdoor ride took place [75,76,77,78]. Two participants reported that they enjoyed the outdoor ride because the route was prescribed. Thus, in riding outdoors, there may have been a restorative effect on cognitive load. In contrast, the indoor ride was daunting because it greatly exceeded the amount of time any of the participants had previously spent cycling indoors, despite the fact that all riders used an indoor bike as part of their regular training programme. In addition, the use of music and/or video is commonplace when cycling indoors, and their absence may have had an impact on the perception of effort [79]. Music has been shown to increase parasympathetic activity [80], which might have reduced the difference in the mental demand score between the indoor ride and the outdoor ride. The protocol for recording HRV may have accelerated recovery as a function of breathing at 0.1 Hz, a frequency known to maximise the resonant frequency of HRV [81] and thus accelerate the parasympathetic response. This may have resulted in a more relaxed state, restoring theta power and HRV to their pre-ride levels and in turn resulting in a similar PVT response. Ultra-distance events typically involve cycling for periods substantially greater than the test duration used in this study (5 h). The limited impact on mental workload found in this study could be because the test duration was too short.

## 5. Conclusions

In the literature on mental workload in cycling, this study is unique in that its conclusions are based on a duration, distance and environment that reflect the milieu of the ultra-distance cyclist. Previous research into mental workload in cycling has tended to be laboratory-based and for shorter durations. Cycling for a period of 5 h, continuously at 65% of FTP, generates a limited measurable increase in mental workload as indicated by the pre and post ride measurement of theta brain wave power, psychomotor vigilance, and the post ride assessment of mental workload reported using the NASA-TLX.

The changes in HRV are commensurate with those expected from exercise but on their own insufficient to attribute them to the effects of mental workload. This contrasts with other modes of transport such as driving or flying, where increases in mental workload over time can lead to errors and increase the likelihood of an accident or injury. The findings from this research suggest that, from a mental workload perspective, activities such as ultra-distance cycling are not demanding.

## Data Availability

The data presented in this study are available on request from the corresponding author. The data are not publicly available due to commitments made in accordance with consent provided by participants.
